# Plants forage for soil patches free of plastic pollution but cannot bag the profits

**DOI:** 10.1038/s41598-023-45662-7

**Published:** 2023-10-28

**Authors:** Benedikt Speißer, Mark van Kleunen

**Affiliations:** 1 Ecology, Department of Biology, University of Konstanz, 78464 Constance, Germany; 2 Zhejiang Provincial Key Laboratory of Plant Evolutionary Ecology and Conservation, Taizhou University, Taizhou, 318000 China

**Keywords:** Ecology, Grassland ecology

## Abstract

Microplastics can affect their surroundings physically and chemically, resulting in diverse effects on plant-soil systems. Similar to other substances (e.g. nutrients and water), microplastics in the environment occur in patches. Such heterogeneous distributions could affect plant responses to plastic pollution. Yet, this has remained untested. We conducted a multispecies experiment including 29 herbaceous plant species and three different microplastic treatments (a control without microplastics, a homogeneous and a heterogeneous microplastic distribution). Based on biomass and root-morphological traits, we assessed how different plastic distributions affect the performance and root-foraging behavior of plants, and whether stronger root foraging is beneficial when microplastics are distributed patchily. Next to general effects on plant productivity and root morphology, we found very strong evidence for root-foraging responses to patchy plastic distributions, with a clear preference for plastic-free patches, resulting in 25% longer roots and 20% more root biomass in the plastic-free patches. Interestingly, however, these foraging responses were correlated with a reduced plant performance, indicating that the benefits of plastic avoidance did not compensate for the associated investments. Our results provide new insights in plant-microplastic interactions and suggest that plants might not just be passively affected by but could also actively respond to environmental plastic pollution.

## Introduction

Since the beginning of the industrial production of plastics in the 1950s, vast amounts of these synthetic polymers have been produced. As a consequence, ever increasing quantities of plastic waste are entering the environment^1,2^. Especially microplastics, particles < 5 mm^3^, might be of major ecological relevance due to various negative influences on ecosystems^4–7^. Although public and scientific awareness of (micro-)plastic pollution was focused on marine systems at first, it has become obvious that terrestrial systems are critically affected as well^8–11^. In fact, the majority of plastic waste is primarily ending up in terrestrial ecosystems^1,2,12^. Consequently, efforts to better investigate potential consequences of plastic pollution, microplastics in particular, on terrestrial ecosystems have increased in recent years. However, although plants constitute a crucial part of terrestrial ecosystems, we are just beginning to understand how plants respond to plastic pollution, and the published findings are partly contradictory^13–17^. In particular, whether plants are just passively affected by microplastics, or if they actively respond to plastic pollution, e.g. by growing away from them, in their direct environment remains unresolved.

Microplastics are ubiquitous in terrestrial ecosystems with soils being a major sink^4,8,10^, and microplastic concentrations in soils vary considerably on the global, regional and local scale^11,12,18^. Accordingly, microplastics may have profound impacts on physicochemical and biochemical soil properties. In fact, microplastics can alter soil bulk density, water holding capacity and water flow^19,20^, organic matter decomposition^21^, as well as carbon and nitrogen cycling^22,23^. In addition to alterations in abiotic soil conditions, interactions with soil biota have been reported as well. For example, earthworms can act as transport agents of microplastics^24^ and can thereby dilute the concentrations of microplastics^25^. Moreover, Huerta Lwanga et al.^26^ showed that earthworm growth and survival can be negatively affected, with potential further implications for soil systems. Furthermore, microplastics can alter the abundance and community composition of microarthropods and nematodes, as well as soil microbial activity^27^. Yet, it is likely that such effects on soil biota also depend on certain characteristics, such as the type of microplastic, as recently shown for soil-bacteria communities^28,29^. An extensive overview of microplastic effects on soil systems is provided in Okoffo et al.^30^.

As plants are rooted in the soil, soil pollution by microplastics is likely to directly (e.g. chemically, physically) or indirectly (via changes in biotic and abiotic soil conditions) affect plants^14^. In fact, both direct and indirect effects of microplastics on plants have already been reported^6^. These effects include changes in plant growth and productivity^17,31,32^, root morphology^15,32^, as well as plant-mycorrhiza interactions^33,34^. However, these patterns often are not unambiguous, and sometimes even in opposite directions, as they depend on a variety of factors. For example, van Kleunen et al.^32^ found a concentration dependency between microplastics in the soil and plant growth, with low concentrations promoting growth and higher concentrations inhibiting growth. Also, microplastics represent a highly heterogeneous group, including particles differing in their chemical composition, shape and size, and microplastic effects on plants can depend on those specific particle properties^35^. In addition, species identity might be important, as species can differ in their response to plastic pollution^16^. Yet, most studies, so far, dealt with a limited number of study species, hampering our ability to draw more general conclusions.

Obviously, due to their sessile nature, individual plants cannot move in order to respond to environmental changes. Instead, plants have developed remarkable strategies, such as a high plasticity of the root system, to optimally cope with beneficial and detrimental soil conditions^36^. The highly plastic root system of plants enables them to respond to abiotic and biotic conditions by selectively growing their roots in certain directions and by proliferating them in certain soil patches, i.e. by performing root foraging^37,38^. Root foraging can be an adaptive strategy for plants in patchy environments, e.g. increasing the capacity to exploit locally abundant resources^39–42^. At the same time, plants also perform root foraging to reduce effects of adverse conditions, such as competition or drought^43,44^, or to avoid potentially toxic or harmful substances^45,46^. As heterogeneity is a general characteristic of soils^47^, microplastics are likely to be heterogeneously distributed in soils as well. Consequently, if microplastics have detrimental effects on plants^6,48^, it is likely that plants could perform root foraging to avoid soil patches with high microplastic concentrations. So far, however, no study has tested whether plants respond to microplastics in soils by root foraging, and if such a response would be of benefit for the plants.

To test if plants respond to patchy microplastic distributions by selectively directing their root growth (i.e. by performing root foraging), and to assess the adaptive value of this response, we performed a multi-species greenhouse experiment. We grew 29 common Central European grassland species under three scenarios: no microplastics in the soil (control), homogeneous microplastic distribution, and heterogeneous microplastic distribution. We assessed various root traits (root biomass, root length, root diameter, specific root length and root branching) in order to test our hypothesis that plants perform root foraging to avoid areas with high plastic concentrations in patchy soils. In addition, to assess whether root foraging is likely to be adaptive, we tested whether intra- and interspecific differences in plant performance were related to foraging responses. In other words, our main question was whether plants can increase their performance by actively avoiding soil patches with high plastic concentrations?

## Materials and methods

### Study species and experimental setup

To investigate the root-foraging responses of plants to heterogeneous microplastic distributions in soils, we conducted a multi-species experiment in a greenhouse of the botanical garden of the University of Konstanz (N: 47°69′19.56′′, E: 9°17′78.45′′). To include a broad range of plant species and to increase the generalizability of the results^49^, we initially selected 45 grassland species. However, due to poor germination of some species, we finally included 29 species belonging to 11 families in the experiment (Supplementary Table 1). To obtain seedlings of similar developmental stages at transplantation, the species were sown at different dates. The species were sown separately in plastic trays filled with potting soil (Einheitserde®, Pikiererde CL P). All trays were placed in a greenhouse with a regular day-night rhythm of approx. 14:10 h. Seeds of all species used in the experiment originated from a commercial seed company (Rieger-Hofmann GmbH, Germany), or from the botanical garden of the University of Konstanz, directly (Supplementary Table 1). No wild plant material was collected or used in the experiment. We confirm that we had the permission by the botanical garden of the University of Konstanz to use the seeds for our study purposes and that our study was carried out in compliance with relevant institutional, national, and international guidelines and legislation.

**Figure 1:**
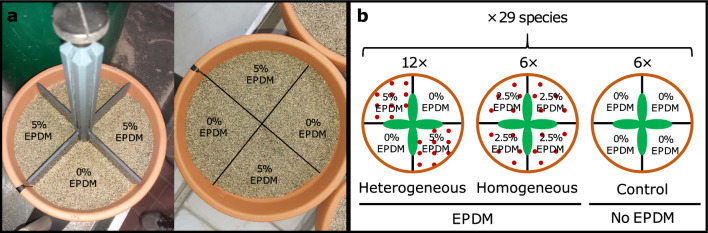
Treatment design for testing the effects of heterogeneous soil-plastic pollution on root-foraging behavior. (**a**) Heterogeneous treatment pots with patches (quarters) with (5%, v:v), and without EPDM (0%). (**b**) Schematic overview across different treatment types. Plants were grown in substrate with heterogeneous plastic distribution (i.e. patches with 5% and 0% (v:v), respectively), homogeneous plastic distribution (2.5%, v:v), or without EPDM granules (0%, control). Note that the divider was removed after filling the pots. There were 12 replicates for each species in the heterogeneous treatment, and 6 replicates for each species in the homogeneous treatment and in the control treatment.

On 10 May 2021, we transplanted the seedlings of 23 of the 29 study species. The remaining six species, which had delayed germination, were transplanted 1 week later. One seedling was placed in the center of each pot, and the pots were placed in a greenhouse, and randomly assigned to fixed positions. To be able to account for differences in the initial size of the seedlings in the statistical analysis, we measured the length and width of the largest leaf and counted the number of leaves per seedling, and we multiplied these three variables to obtain a proxy for the total leaf area. To reduce potential effects due to pot position, the positions of pots were re-randomized 3 weeks after the start of the experiment. To ensure sufficient water and nutrient supply, plants were watered regularly with tap water, and we fertilized them weekly during the first 5 weeks of the experiment (1‰ Universol® blue oxide, ICL SF Germany & Austria, Nordhorn, Germany). To avoid possible plastic-pollution effects of commonly used plastic pots, we used clay pots (1 L) instead. To ensure that the roots could easily be washed free from substrate, we used a 1:1 (v:v) sand-vermiculite mixture as substrate. To test the plant responses to patchy plastic distributions, we created three treatments, i.e. control (without any plastics), heterogeneous, and homogeneous microplastic distribution (Fig. 1). The homogeneous-treatment pots were filled with a substrate-microplastic mixture containing 2.5% (v:v) homogeneously distributed granules (0.5–2.5 mm) of the synthetic rubber ethylene propylene diene monomer (EPDM, Resedagrün RAL 6011, GranuElastic Höfer & Stankowska GbR). EPDM granules were chosen because they are frequently used in artificial turfs (e.g. football fields), from which they easily spread into the surrounding vegetation, and because they were shown to affect plant growth in a previous experiment^32^. EPDM is produced and used for outdoor applications, and highly resistant against abrasion and UV-degradation. The stability and durability of EPDM, therefore, makes it highly unlikely that any recognizable degradation or decomposition occurred during the course of the experiment. For the heterogeneous treatment, we divided the pots into four quarters, of which two opposing quarters were filled with plastic-free substrate (control patches), and the two other ones were filled with a substrate-plastic mixture containing 5% EPDM granules (microplastic patches; Fig. 1). So, pots in the homogeneous and heterogeneous treatments contained the same absolute amounts of microplastics, and only differed in spatial distribution and local concentration of the granules.

To ensure accurate plastic concentrations, we prepared the substrate-plastic mixtures individually for each pot (homogeneous treatment) or each pot quarter (heterogeneous treatment). To make sure that the plastic addition in the heterogeneous treatment was constrained to the assigned patches, we used pot dividers that were specially designed and manufactured for the experiment, enabling us to fill each pot quarter individually (Fig. 1a). After filling the respective pot, the divider was carefully removed from the pot, so that roots could freely grow from one patch into the others. In order to compare the patches of the heterogeneous treatment to the homogeneous control and plastic treatment, we also assigned a priori two quarters of the homogeneous treatments as dummy control patches and two quarters as dummy microplastic patches. We checked the distribution of plastic particles at the end of the experiment and did not observe migration of particles from the plastic patches to the plastic-free patches in the heterogeneous treatment. For each species, there were six replicates for the control and homogeneous treatment, respectively. For the heterogeneous treatment, to increase the statistical power for analyzing how variation in foraging responses relates to performance within species, we included twelve replicates per species. Consequently, the experiment comprised a total of 696 pots (29 species × [[2 treatments × 6 replicates] + [1 treatment × 12 replicates]]).

### Harvest and measurements

To reduce potential pot-limitation effects due to the confined soil volume^50^, we limited the growth period to 6 weeks and harvested the plants starting on 21 June 2021. The aboveground parts were cut directly at the soil surface, cleaned from substrate residues, stored in paper bags, and dried at 70 °C for at least 72 h. The roots were harvested separately from each of the previously assigned pot quarters—corresponding to true (heterogeneous treatment) or dummy control and microplastic patches (control and homogeneous treatment)—and the central root parts. To do so, we used a soil corer (diameter: 2 cm) with four knife blades on its outside at 90° angles to cut the substrate and the roots (Supplementary Fig. 1). Subsequently, the substrate was carefully washed off the respective root parts. For each pot, a priori, one plastic (or dummy plastic) and one control (op dummy control) quarter were randomly assigned for the root-morphology analysis. Therefore, the roots of these quarters were stored in water-filled plastic tubes (Supplementary Fig. 1) at approx. 10 °C. Later, we digitalized these root parts using a modified flatbed scanner and subsequently analyzed their morphology using the WinRhizo™ Pro imaging software (Regent Instruments, Canada). All root samples (five samples per plant) were separately stored and dried in paper bags as described above and subsequently all plant samples (shoot and root samples) were weighed. To test for potential effects of microplastics on the soil moisture, we measured the water content for each pot on the first day of the harvest by performing one measurement per pot at a random position using a HH2 moisture meter equipped with a WET-2 sensor (Delta-T devices Ltd, Cambridge, UK).

### Statistical analysis

All statistical analyses were performed in R 4.1.2^51^. As almost all individuals of *Leucanthemum ircutiatum* (18 out of 24), and all individuals of *L. vulgare* died during the experiment, we excluded these two species, resulting in 27 species in the analysis.

#### Plant biomass

To assess potential effects of soil-microplastic pollution on the biomass production of plants, we fitted linear mixed-effects models using the *lme* function of the “nlme” package^52^. We ran separate models including total plant biomass, shoot biomass, root biomass and root-weight ratio (root biomass divided by total plant biomass) as response variables and microplastic treatment (control, homogeneously distributed, heterogeneously distributed) as fixed effects. To account for variation in initial plant size, we added initial leaf area (scaled and centered, log_e_-transformed) as covariate. To improve normality of residuals, total biomass, root biomass and shoot biomass were cube-root transformed. To account for non-independence of replicates of the same species and for phylogenetic relatedness, we included species nested within family as random factors. Further, we initially included random slopes for family and species with respect to the microplastic treatment but removed them if they did not improve the model fit (i.e. if they did not reduce the AIC). To improve homoscedasticity, we added variance structures for species using the *varIdent* function in the “nlme” package^52^. To assess fixed-factor effects, we used  log-likelihood-ratio tests^53^. To further compare effects on the root-weight ratio among the different treatments, we used pairwise post-hoc comparisons using the “emmeans” package^54^.

#### Root morphology and foraging responses

Root-morphology and foraging-response analyses were performed based on the data of the root-morphology samples described above (i.e. the two root samples per plant).

To test whether microplastics in the soils affected root morphology in general, we directly analyzed the root-morphology traits root length, root diameter, link length (as a measure for the branching frequency) and specific root length. To do so, we used trait values based on both samples of each plant as response variable in separate linear mixed-effects models including treatment as explanatory variable. That is, the sum of both samples for root length, or the average of both samples for root diameter and link length weighted by the lengths of the respective root samples. The combined specific root length was calculated as the total root length of both samples divided by the total root dry weight of both samples. General treatment effects and specific differences among treatments were assessed as described above.

To test whether the plants in our experiment responded to heterogeneous microplastic distributions in the soil by changing their root growth and morphology (i.e. performing root foraging), we assessed within-pot differences in root-mass distribution and root morphology, and compared them among the different treatments. First, we directly analyzed the root traits mentioned above at the patch level. We analyzed the patch-level differences using linear mixed-effects models including the respective root trait as response variable. Data for root diameter, average link length and specific root length was natural-log transformed, data for root biomass was cube-root transformed and data for total root length was square-root transformed. Treatment and patch, i.e. (dummy) control and (dummy) microplastic, and their interaction were included as fixed effects. To account for non-independence of samples from the same pot, we included pot nested within species nested within family as random effects. Fixed-factor effects were assessed as described above.

In addition, to obtain a measure for the root-foraging response, we calculated a foraging index^41,42^ for the above-mentioned traits. This was done for the treatment with heterogeneously distributed microplastics, but also for the control treatment and the treatment with homogeneously distributed microplastics, using the a priori assigned dummy patches. For root biomass, we used the mean values of both (dummy) control and (dummy) microplastic patches. For root length, root diameter and root biomass, we calculated the foraging index (FI) for each individual plant as:

1 $$FI_{T} = \frac{{T_{c} - T_{MP} }}{{T_{c} + T_{MP} }}$$

Here, *T*_*C*_ represents the trait value for the control patch and *T*_*MP*_ the trait value for the microplastic patch. That is, the foraging index (*FI*) for a certain trait (*T*) is the quotient of the difference between the trait value in the (dummy) control patch (*T*_*C*_) and the trait value in the (dummy) microplastic patch (*T*_*MP*_) of the same pot divided by the sum of both patches^41,42^. This way, higher values for the foraging index indicate a higher trait value in the control patch and thus a positive foraging response, i.e. a plastic-avoidance response. Conversely, as increased values for specific root length and longer average link length indicate more absorptive but less branched roots, as a response to suboptimal soil conditions^55^, we expected these trait values to increase in the presence of plastics. Therefore, we calculated the foraging index for these traits as:

2 $$FI_{T} = \frac{{T_{MP} - T_{C} }}{{T_{C} + T_{MP} }}$$

Hence, by subtracting the trait value of the (dummy) control patch from that of the (dummy) microplastic patch, a higher foraging-index value again indicates a positive root-foraging response.

We then ran linear mixed-effects models, as described above, separately for each trait-foraging index as response variable and microplastic treatment as explanatory variable. Subsequent to the linear mixed-effects models, we assessed fixed-factor effects using log-likelihood-ratio tests and did pairwise post-hoc comparisons for the treatment effect.

#### Adaptivity of foraging response

Avoidance of high-concentration microplastic patches by increased root foraging could reduce negative microplastic effects on plants and thus increase the average performance of plant species or individual plants. To test this assumption—stronger root-foraging responses increase plant performance, among species and among individuals within species—we tested whether plants with stronger root foraging (i.e. higher FI_T_-values) also performed better in terms of biomass production^42^. We assessed the adaptivity of root foraging in the context of plastic pollution based on the data of plants in the heterogeneous treatment only (n = 316). To evaluate the adaptive value of root foraging among and within species, we ran linear mixed-effects models with within-species mean centering^41,56^. To do so, we calculated the species average for each FI_T_ and the respective individual plant deviations from the average of the respective species the individual plant belongs to. We then fitted linear mixed-effects models including cube-root transformed total plant biomass as response variable, average species FI_T_ and individual plant deviations as fixed continuous variables. Fixed-term effects were assessed using log-likelihood-ratio tests. We included family and species nested within family as random effects.

## Results

Given the long-standing discussions around the arbitrary p-value cutoff, we followed the recommendations of Muff et al.^57^, and wrote the results in the language of evidence.

### Biomass

**Figure 2:**
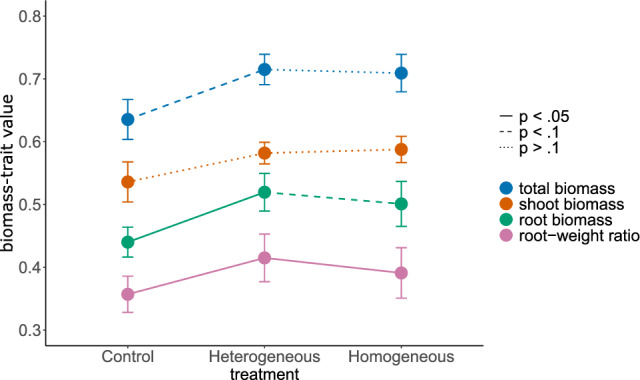
Total biomass, shoot biomass, root biomass and the root-weight ratio of plants in the different microplastic treatments (control, heterogeneous, homogeneous), based on pot-level data. Colored points represent model estimates for total plant biomass (blue), shoot biomass (orange), root biomass (green) and root-weight ratio (magenta). Error bars represent the respective standard errors. Biomass traits were measured in grams. To improve normality of residuals, total biomass, root biomass and shoot biomass were cube-root transformed. For root biomass and root-weight ratio, different line types indicate statistical significance for the respective post-hoc comparisons (Supplementary Table 4). There was weak evidence for overall microplastic effects on total biomass (*p* = 0.056) and no evidence for effects on shoot biomass (*p* = 0.3).

We first assessed overall effects of homogeneous and heterogeneous microplastic distributions in the soil on plant biomass as a measure of plant performance. There was weak evidence that microplastics increased total plant biomass, irrespective of the homogeneous or heterogeneous distribution (+ 11.2% and + 12.04%, respectively; *p* = 0.056; Supplementary Table 2; Fig. 2). The increase in total plant biomass was driven by a general increase of root biomass in both treatments with microplastics (homogeneous: + 12.7%, heterogeneous: + 16.5%), rather than by changes in shoot biomass (*p* = 0.298; Supplementary Table 2; Fig. 2). This pattern is also reflected in a higher root-weight ratio of plants grown in the presence of microplastics (*p* = 0.028; Supplementary Table 2; Fig. 2). Further, compared to the homogeneous plastic distribution, the effect of microplastics on the root-weight ratio was even stronger when they were distributed heterogeneously (+ 6.01%; p < 0.001; Supplementary Table 2; Fig. 2; Supplementary Table 4).

### Root morphology

To evaluate general effects of microplastics on root morphology, we first assessed the effects on the different traits based on the average or sum of both samples for each individual, as explained in the Materials and Methods. Overall, plants grown in the presence of microplastics tended to produce longer roots (homogeneous: + 18.1%, heterogeneous: + 20.02%) compared to plants grown in plastic-free substrate. Yet, there was only weak evidence for this trend (*p* = 0.091). In contrast, there was very strong evidence that plants produced thicker roots in the presence of microplastics (*p* < 0.001; Supplementary Table 3), and this effect was stronger in the heterogeneous (+ 6.2%) compared to the homogeneous treatment (+ 3.7%; pairwise comparison *p* = 0.025; Supplementary Table 5). In line with the generally thicker roots, plants in both plastic treatments had a lower specific root length, irrespective of the distribution, compared to plants grown in the absence of plastic (homogeneous: − 1.5%, heterogeneous: + 1.8%; *p* = 0.038; Supplementary Table 3). In addition, there was moderate evidence for a higher root-branching frequency in both plastic treatments, indicated by shorter average link lengths (homogeneous: − 35.3%, heterogeneous: − 42.3%; *p* = 0.031; Supplementary Table 3). However, there was no evidence for differences between the homogeneous and heterogeneous plastic treatments (pairwise post-hoc comparison *p* = 0.211; Supplementary Table 5).

### Foraging response

**Figure 3:**
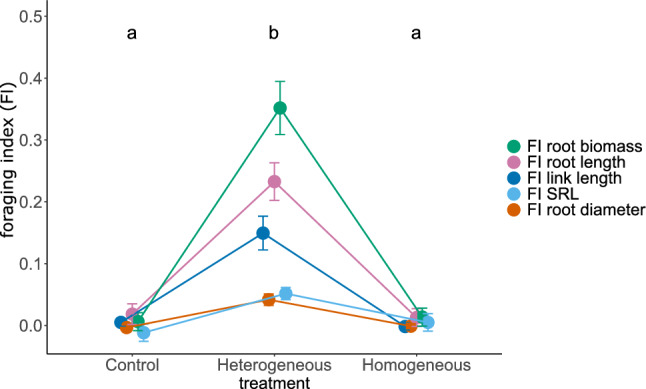
Root-foraging responses to the control, and the homogeneous and heterogeneous microplastic distributions, calculated as foraging index (see Eqs. 1 and 2). Colored points represent model estimates for the foraging index for root biomass (green), root length (magenta), average link length (dark blue), specific root length (SRL; light blue) and average root diameter (orange). Error bars represent the respective standard errors. Letters indicate differences between control, heterogeneous and homogeneous treatments based on pairwise post-hoc comparisons.

**Figure 4:**
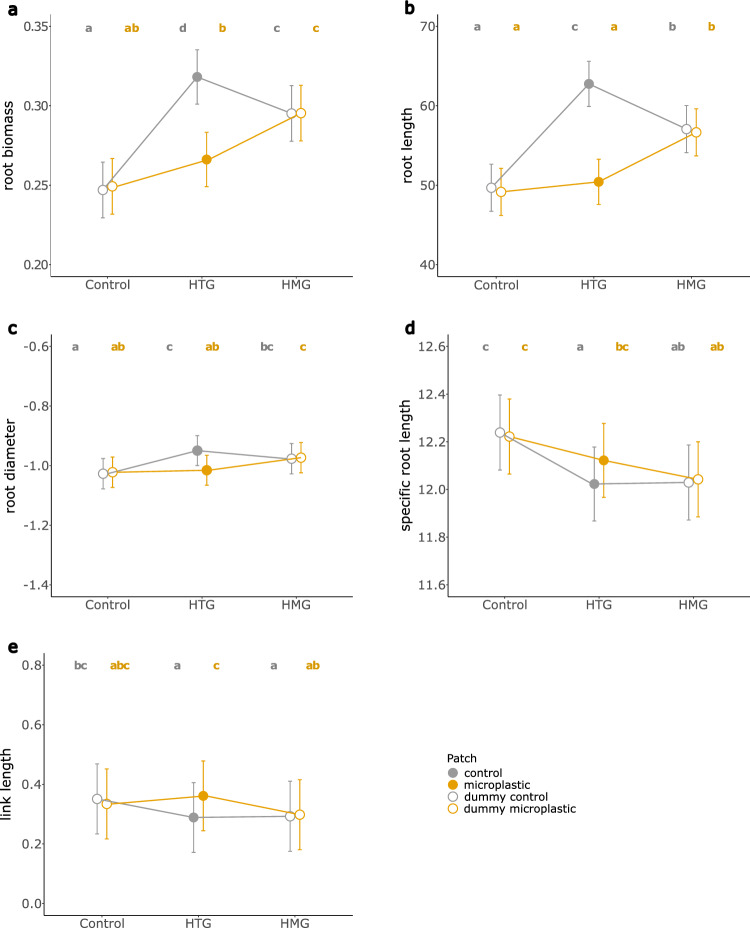
Root-morphology-trait values in the patches of the different microplastic treatments, based on patch-level data. Colored symbols represent post-hoc estimates for the respective traits. Note that, in the experimental setup, only in the heterogeneous treatment (HTG) microplastic (gold) and control (grey) patches really differed from each other. In the control and the homogeneous (HMG) treatment, they represent dummy patches, indicated by the open symbols, that in reality did not differ from one another (Fig. 1). Error bars represent the respective standard errors. Root biomass (**a**) was measured in g and was cube-root transformed, root length (**b**) was measured in mm and was square-root transformed, root diameter (**c**) was measured in mm, specific root length (**d**) was calculated in mm/g, and average link length (**e**) was measured in mm. Root diameter, specific root length and average link length were log_e_-transformed. Letters indicate differences based on pairwise post-hoc comparisons. Groups sharing a letter do not significantly differ from each other.

Based on the root-trait differences between dummy-plastic and dummy-control patches in the control treatment and the treatment with homogeneously distributed microplastics, and between real plastic and control patches in the treatment with heterogeneously distributed microplastics, we assessed the root-foraging response of plants to patchy plastic distributions, represented by a foraging index (FI) for the respective trait (Eqs. 1 and 2). As expected, for the control and the homogeneous plastic treatment the calculated FI values for each trait were always close to zero, indicating no foraging responses in these treatments. In contrast, there was strong evidence for root foraging, i.e. plastic avoidance in the heterogeneous plastic treatment for all measured traits (Table 1; Fig. 3). That is, while there were no patch differences in the control and the homogeneous plastic treatment, there were clear differences between the plastic and the plastic-free patches in the heterogeneous plastic treatment (Supplementary Table 6; Fig. 4). Overall, there were more (+ 19.5%), longer (+ 24.5%) and thicker (+ 6.8%) roots in the plastic-free patches of the heterogeneous plastic treatment. Furthermore, the roots in the plastic-free patches were more branched, indicated by a shorter average link length in the plastic-free patches (− 20.1%).

**Table 1:** Results of linear mixed-effects models testing root-foraging responses due to different microplastic (MP) distribution treatments.

		FI root biomass			FI SRL			FI root length			FI root diameter			FI link length	
*Fixed effects*	df	LLR	p		LLR	p		LLR	p		LLR	p		LLR	p
Initial leaf area	1	0.106	0.745		0.122	0.726		2.385	0.123		0.649	0.42		1.175	0.278
MP treatment	2	23.609	** < 0.001**		14.349	** < 0.001**		28.343	** < 0.001**		12.011	**0.0025**		18.114	** < 0.001**
*Random effects*		SD			SD			SD			SD			SD	
		Control	HTG	HMG	Control	HTG	HMG	Control	HTG	HMG	Control	HTG	HMG		
Family		0.015	0.119	3.75e^−10^	0.483	0.12	0.169	11.276	19.383	21.162	0.004	0.031	0.006	0.132	
Species		–			0.302	0.278	0.247	17.758	13.783	16.39	–			0.107	
Residual		0.188			0.423			13.519			0.056			0.216	

Root-foraging responses were assessed based on the calculated foraging index (FI) for the respective trait (Eqs. 1 and 2). All models included random intercepts for species nested within families, and the models for root biomass, specific root length (SRL), total root length and root diameter additionally included random slopes with regard to the heterogeneous (HTG) and homogeneous (HMG) microplastic treatments, relative to the control. Fixed-factor effects were assessed using log-likelihood ratio tests^53^. Log-likelihood ratios (LLR) are approximately χ^2^-distributed. *p* values < 0.05 are indicated in bold. HTG refers to the heterogeneous plastic treatment, HMG to the homogeneous.

### Adaptivity of foraging responses

**Figure 5:**
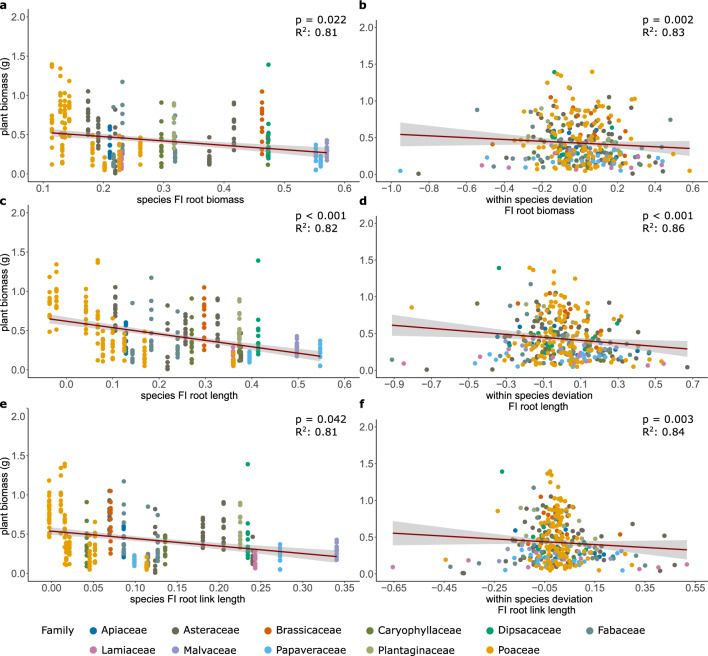
Adaptive value of root-foraging responses regarding root biomass (**a**, **b**), root length (**c**, **d**) and root branching (measured as average link length; **e**, f**)** among (**a**, **c**, **e**) and within (**b**, **d**, **f**) species. Species foraging index (FI) values refer to the average FI of each species. Within species deviation FI values refer to the deviation of individual plants from the species average FI. Colors represent the families the study species belong to. *p* values were obtained using log-likelihood-ratio tests, R^2^ values represent the conditional R^2^ of the respective model.

To evaluate whether root-foraging responses (i.e. microplastic avoidance) had an adaptive value for the plants, we assessed how the strength of the foraging response affected plant performance (i.e. plant productivity) among and within plant species in the heterogeneous treatment. We found that for root biomass, root length and average link length (root branching) stronger foraging responses (higher FI) were correlated with lower plant performance, both among and within species (Table 2; Fig. 5). That is, plant species, or individuals within a species, that had stronger foraging responses tended to produce less biomass than species, or individuals of the same species, with weaker foraging responses.

**Table 2:** Results of linear mixed-effects models testing the adaptive value of root-foraging responses (FI values) to patches with and without microplastics in the heterogeneous microplastic-distribution treatment among and within species. The response variable in all models was total biomass.

		FI root biomass		FI SRL		FI root length		FI root diameter		FI link length	
*Fixed effects*	df	LLR	p	LLR	p	LLR	p	LLR	p	LLR	p
Initial leaf area	1	79.426	** < 0.001**	71.167	** < 0.001**	76.098	** < 0.001**	70.64	** < 0.001**	75.305	** < 0.001**
Among Species	1	5.271	**0.022**	1.841	0.175	11.706	** < 0.001**	0.193	0.66	4.142	**0.042**
Within Species	1	9.165	**0.002**	0.03	0.863	14.888	** < 0.001**	0.63	0.428	8.619	**0.003**
*Random effects*		SD		SD		SD		SD		SD	
Family		0.0016		0.0366		0.0017		0.0262		0.0021	
Species		0.0934		0.0952		0.0823		0.101		0.0962	
Residual		0.06		0.0648		0.0534		0.0661		0.0589	

Fixed-factor effects were assessed using log-likelihood ratio tests^53^. Log-likelihood ratios (LLR) are approximately χ^2^-distributed. *p* values < 0.05 are indicated in bold. The models included random intercepts for species nested within families.

## Discussion

Although research on ecological effects of microplastics only recently started to also consider terrestrial ecosystems^10^, there is increasing evidence for clear effects on soils and plants^17,21,35,58^. In this context, our multispecies experiment adds new insights but also raises new questions. Our findings indicate that pollution by microplastics per se, as well as how the particles are distributed in the soil can have diverse effects on overall plant growth and root morphology. For example, microplastic addition resulted in a higher absolute and relative root biomass, an effect which was stronger in the heterogeneous than in the homogeneous microplastic-distribution treatment (Supplementary Table 4; Fig. 2). Furthermore, heterogeneous distributions of microplastic particles in the soil had profound effects on the root-growth behavior of the plants. In line with our hypothesis, plants showed clear foraging responses by preferring the plastic-free soil patches over the patches containing microplastics (Supplementary Table 6; Figs. 3 and 4). This suggests that the plants tended to avoid microplastics in the soil, despite the generally positive effect on plant productivity. Our results thus show that plants respond to patchy plastic distributions in the soil by changing their root growth behavior, which could have further implications for plant growth but also indicates that plants might be able to partially evade highly polluted soil patches.

Generally, the plants grown in substrate containing microplastics, in both the heterogeneous and homogeneous treatment, tended to produce more biomass than the plants in the control treatment (Supplementary Table 2; Fig. 2). Both positive^16^ and negative^17^ microplastic effects on plant biomass have been reported before. Yet, they seem to be highly context dependent and can inter alia differ among species^16^. However, only few studies testing microplastic effects on plants included multiple species^59^, impeding more general conclusions. In our multi-species study, microplastics tended to increase total plant biomass, when averaged across all 27 species, but also here, the effect differed among species (treatment:species-interaction *p* < 0.001, Supplementary Fig. 2).

In addition to species identity, also the material the plastic particles consist of plays a central role in shaping their effects^6,60^. This is because different plastic types differ in properties, such as their chemical composition, the presence of additives and other chemical characteristics, which can influence how the plastic particles affect and interact with their surroundings. Especially the release of chemical contaminants—such as additives or hydrophobic organic pollutants via leaching and sorption–desorption processes—could be a critical determinant of the ecological effects of microplastics^6,61^. For EPDM, a clear dose–response relationship was shown before. That is, higher concentrations (5% and higher) had strong negative effects on plant growth, whereas lower concentrations, comparable to the concentration used in our experiment, slightly improved plant growth^32^. This indicates that negative chemical effects of EPDM at higher concentrations outmatched potential positive physical effects. Indeed, plastic particles in the soil could also improve plant growth by promoting the formation of soil pores and thereby facilitating root growth^62^. Such positive physical effects on soil structure could also explain the increased root growth we observed in the homogeneous microplastic treatment (Fig. 4).

The heterogeneous microplastic treatment clearly affected plant productivity by increasing overall root growth, without clear effects on shoot biomass, thus resulting in a higher root-weight ratio compared to both the control and homogeneous microplastic treatment (Supplementary Table 4; Fig. 2). However, these differences do not show the full picture of heterogeneity effects, as they just display the overall effects at the pot level. In more detail, the overall differences between the heterogeneous microplastic treatment and the control and homogeneous treatment were brought about by patch specific changes in root biomass, root length and diameter, average link length and specific root length (Supplementary Table 7; Fig. 4). These changes are reflected in the high foraging-index values (FI) for these traits, indicating a strong foraging response of plants grown in soils with patchily distributed microplastics (Fig. 3). Yet, it is not completely clear what is driving these responses.

The high FI values (Fig. 3) in the plastic patches of the heterogeneous treatment (5% plastic concentration) compared to the homogeneous treatment (2.5% plastic concentration) could indicate direct negative effects of higher plastic concentrations as indicated by van Kleunen et al.^32^. All these values indicate that the plants shift their belowground resource investments towards the roots in the plastic-free patches, resulting there in more persistent (higher root diameter), more branched (shorter link length), and overall more roots (increased root length and biomass; Fig. 4). Next to direct effects, indirect effects could play an important role as well. For example, it has been proposed that microplastics could affect the soil C:N ratio^14^ and nitrogen cycling^63^ by altering enzymatic activities^64^, which could affect nutrient availability and uptake by plants. Indeed, Ingraffia et al.^65^ recently reported that polyester microfibers reduced the nitrogen uptake of maize plants by about 30%. In addition, microplastics could also reduce soil moisture and thus the amount of water that is available to plants^66^. In fact, in our experiment, soil moisture was clearly lower in both microplastic treatments compared to the plastic-free substrate (-9.3%, *p* = 0.016), possibly due to the hydrophobic properties of the used EPDM particles^67^. Plants are known to be able to respond to heterogenous soil conditions by means of root foraging. This is especially well established in the context of nutrient heterogeneity^37,39,68^, where plants can increase their performance by better exploiting nutrient rich patches^42,69^. Likewise, plants respond in similar ways to differences in water availability in soils^70^. This could also be a part of the explanation for the reduced root diameter and increased SRL in the patches with microplastics of the heterogeneous treatment. Thin roots with a high SRL, due to their increased surface-volume ratio, have a better absorption capacity, making them beneficial under conditions of reduced resource availability^41,71^. Consequently, the root-foraging responses observed in our experiment could be caused by direct toxicity effects of microplastics, altered water and nutrient availability or, most likely, an interplay of these factors.

It is generally assumed that the ability of plants to perform root foraging in heterogeneous environments should increase plant performance, i.e. has an adaptive value. However, few studies have explicitly tested this, and those that did mainly focused on positive factors such as nutrient availability^41,42^. In those studies, root foraging towards high nutrient patches was found to be associated with better plant performance. In contrast, it is not clear whether plants also gain benefits by performing root foraging to avoid or escape detrimental conditions. The major difference between both types of foraging response is that in the first case foraging results in an increased resource availability, whereas the second case rather restricts plants in their capacity to effectively exploit their soil environment. That is, root foraging, as other forms of phenotypic plasticity, comes with costs and limits^72–75^, confining the potential benefits.

In our experiment, stronger root foraging of plants grown in heterogeneous soil conditions was not associated with better plant performance. Quite the opposite, stronger foraging in terms of root biomass, root length and average link length was correlated with lower plant performance both among and within species (Table 2; Fig. 5). That is, species and individuals with stronger foraging responses (higher FI) performed worse than other species or individuals of the same species with weaker foraging responses. However, there might be differences among plant functional groups, indicated by significant interactions between the foraging response (species FI) and functional group affiliation, influencing the effects on plant performance (Supplementary Fig. 3). Still, the generally negative relationship between stronger foraging response and reduced plant performance could indicate that, in the case of patchy plastic distributions, the investments the plant has to make to perform root foraging exceed the potential benefits^42,69^, i.e. there is a limit of plasticity^74^. On the one hand, a stronger foraging response might have reduced negative effects of higher plastic concentrations. On the other hand, however, it also reduced the effective soil volume occupied by plant roots. That is, the plants clearly increased their root growth in the plastic-free patches (Fig. 4). This not only reduced the absolute soil volume from which the plants could extract water and nutrients but might also have led to a faster nutrient depletion in those patches. Another potential explanation for the lower performance of plants with higher FI values could be that root foraging is not an appropriate response to plastic pollution. Plants sense their surrounding in various ways and respond to different environmental cues via sophisticated pathways^76–80^. However, plastic pollution and microplastics in the soil are evolutionarily novel to plants, potentially resulting in an information reliability limit^73^. That is, although plants might be able to sense microplastics in the soil based on mechanical or chemical cues, this might not lead to adequate responses but could result in maladapted phenotypes due to the just recent emergence of this particular environmental contaminant. Future studies should test whether plants might evolve adaptive responses when exposed to microplastic pollution for several generations.

In natural environments, effects of root foraging due to heterogeneous plastic distribution might differ, as additional factors can come into play as well. To be able to explicitly test for effects of heterogeneous plastic distribution, in our experiment, the local plastic concentration was the only heterogeneity factor, while keeping other soil characteristics uniform. In nature, soil patches might not only differ in terms of plastic concentration but soil properties could differ as well, which could influence microplastic effects^62^. In addition, toxicity effects of microplastic particles might be more pronounced under natural conditions due to several mechanisms related to aging and weathering, including the leaching of additives and other chemical contaminants, transfer and accumulation of pollutants such as heavy metals or hydrophobic organic contaminants^61,81–84^. Further, also adverse physical effects could play a bigger role due to the disintegration of plastic fragments resulting in nanoparticles that can permeate cell membranes and directly affect plants in negative ways^59,60^. Accordingly, stronger toxicity effects could increase the benefits of plastic avoidance due to root foraging. On the other hand, microplastics might affect competition among plants^32^. If co-occurring species respond similarly to heterogeneous plastic distributions, this could result in stronger competition in less polluted patches, reducing potential benefits of plastic avoidance. Therefore, different environmental conditions in natural habitats could be important modulators, affecting both intensity as well as effects of root foraging due to heterogeneous plastic distributions.

Our study provides clear evidence for potentially far-reaching effects of microplastic pollution on plants. In addition, for the first time, we show that plants respond to patchy plastic distributions in soils by performing root foraging, clearly preferring plastic-free soil patches. However, our results also suggest that such responses are not necessarily beneficial for plants, potentially due to limits associated with root foraging. Differing conditions in natural habitats might affect the balance between limits and benefits in either way. Therefore, investigating root-foraging responses under more natural conditions could provide further interesting insights beyond the ones provided by our study.

### Supplementary Information

Supplementary Information.

## Data Availability

All data used for the analyses was obtained from the experiment and is available at https://doi.org/10.6084/m9.figshare.23266661.v1.
