# Supplementary material for: Plants forage for soil patches free of plastic pollution but cannot bag the profits
Source: Sci Rep. 2023 Oct 28;13:18506. doi: 10.1038/s41598-023-45662-7 (PMC10613303; doi:10.1038/s41598-023-45662-7)
Supplement: Supplementary file 1 — Supplementary Information. [file 41598_2023_45662_MOESM1_ESM.pdf]

Supporting information

Plants forage for soil patches free of plastic pollution but cannot bag the profits

Benedikt Speißer[1]*, Mark van Kleunen[1,2]

[1]Ecology, Department of Biology, University of Konstanz, 78464 Konstanz, Germany

[2]Zhejiang Provincial Key Laboratory of Plant Evolutionary Ecology and Conservation, Taizhou University, Taizhou 318000, China

*corresponding author

Contact: benedikt.speisser@uni-konstanz.de, Phone: +49 7531 4145

Supplementary Table 1 Detailed information about the study species used in the experiment.

Species	Family	Growth form	Status	Commonness[†]
Agrostis capillaris[1] L.	Poaceae	perennial grass	native	common
Anthoxanthum odoratum[1] L.	Poaceae	perennial grass	native	common
Brassica nigra[2] (L.) W.D.J Koch	Brassicaceae	annual forb	neophyte	regularly
Bromus hordeaceus[1] L.	Poaceae	annual grass	native	common
Bromus sterilis[2] L.	Poaceae	annual grass	native	common
Centaurea cyanus[2] L.	Asteraceae	annual forb	native	common
Centaurea jacea[1] L.	Asteraceae	perennial forb	native	common
Cynosurus cristatus[2] L.	Poaceae	perennial grass	native	common
Dactylis glomerata[1] L.	Poaceae	perennial grass	native	common
Daucus carota[2] L.	Apiaceae	biennial forb	native	common
Festuca rubra[1] L.	Poaceae	perennial grass	native	common
Gnaphalium uliginosum[2] L.	Asteraceae	annual forb	native	common
Knautia arvensis[1] (L.) Coult	Asteraceae	perennial forb	native	common
Leontodon hispidus[1] L.	Asteraceae	perennial forb	native	common
Leucanthemum vulgare[1] Lam.	Asteraceae	perennial forb	native	common
Leucanthemum ircutianum[1] Turcz. Ex DC.	Asteraceae	perennial forb	native	common
Lotus corniculatus[1] L.	Fabaceae	perennial forb	native	common
Malva moschata[1] L.	Malvaceae	perennial forb	native	common
Papaver dubium[2] L.	Papaveraceae	annual forb	native	common
Papaver rhoeas[2] L.	Papaveraceae	annual forb	native	common
Plantago lanceolata[1] L.	Plantaginaceae	perennial forb	native	common
Poa angustifolia[1] L.	Poaceae	perennial grass	native	common
Poa pratensis[1] L.	Poaceae	perennial grass	native	common
Prunella vulgaris[1] L.	Lamiaceae	perennial forb	native	common
Scorzoneroides autumnalis[1] (L.) Moench *(ex Leontodon autumnalis)*	Asteraceae	perennial forb	native	common
Silene flos-cuculi[1] (L.) Clairv. *(ex Lychnis flos-cuculi)*	Caryophyllaceae	perennial forb	native	common
Silene vulgaris[2] (Moench) Garcke	Caryophyllaceae	perennial forb	native	common
Trifolium pratense[1] L.	Fabaceae	perennial forb	native	common
Vicia sativa[1] L.	Fabaceae	annual forb	archaeophyte	common

[†]Refers to species commonness in Germany, based on data from FloraWeb (https://www.floraweb.de/)

[1]Seeds were obtained from Rieger-Hofmann GmbH, Germany

[2]Seeds originated from the botanical garden of the University of Konstanz

Supplementary Table 2 Results of linear mixed-effects models testing the overall effects of microplastic (MP) distribution treatments (control, heterogeneous, homogeneous) on biomass production and root allocation. Fixed-factor effects were assessed using log-likelihood ratio tests (Zuur et al., 2009). Log-likelihood ratios (LLR) are approximately χ^2-distributed. P values < 0.1 are indicated in italics, values < 0.05 are indicated in bold. The model included random intercepts (Control) and random slopes with regard to the heterogeneous (HTG) and homogeneous (HMG) microplastic treatments for species nested within families.

		Total biomass		Root biomass		Shoot biomass		Root-weight ratio	
Fixed effects	df	LLR	p	LLR	p	LLR	p	LLR	p
Initial leaf area	1	172.871	**< 0.001**	139.245	**< 0.001**	167.632	**< 0.001**	14.697	**< 0.001**
MP treatment	2	5.775	*0.056*	11.619	**0.003**	2.418	0.298	12.697	**0.002**

Random effects		SD			SD			SD			SD	
	Control	HTG	HMG	Control	HTG	HMG	Control	HTG	HMG	Control	HTG	HMG
Family	0.051	0.088	0.096	0.036	0.042	0.057	0.069	0.079	0.089	0.068	0.042	0.048
Species	0.132	0.094	0.106	0.101	0.099	0.109	0.114	0.061	0.073	0.094	0.075	0.079
Residual		0.067			0.056			0.058			0.064	

Supplementary Table 3 Results of linear mixed-effects models testing the overall effects of microplastic (MP) distribution treatments (control, heterogeneous, homogeneous) on root morphological traits. Fixed-factor effects were assessed using log-likelihood ratio tests (Zuur et al., 2009). Log-likelihood ratios (LLR) are approximately χ^2-distributed. P values < 0.1 are indicated in italics, values < 0.05 are indicated in bold. All models included random intercepts for species nested within families, and the models for specific root length (SRL), total root length and average link length additionally included random slopes with regard to the heterogeneous (HTG) and homogeneous (HMG) microplastic treatments, relative to the control.

		SRL			Root length		Root diameter			Link length	
Fixed effects	df	LLR	p	LLR		p	LLR	p	LLR		p
Initial leaf area	1	37.608	**< 0.001**	62.905		**< 0.001**	60.81	**< 0.001**	35.49		**< 0.001**
MP treatment	2	6.553	**0.038**	4.787		*0.091*	50.68	**< 0.001**	6.97		**0.031**
Random effects		SD			SD		SD			SD	
	Control	HTG	HMG	Control	HTG	HMG			Control	HTG	HMG
Family	0.483	0.12	0.169	11.276	19.383	21.162	0.135		0.457	0.176	0.144
Species	0.302	0.278	0.247	17.758	13.783	16.39	0.096		0.269	0.06	< 0.001
Residual		0.423			13.519		0.112			0.412	

Supplementary Table 4 Pairwise post-hoc comparisons of the different microplastic distributions regarding plant productivity, obtained using the "emmeans" package (Lenth, 2021). HTG refers to the heterogeneous plastic treatment, HMG to the homogeneous plastic treatment and CTRL to the control treatment.

	Root biomass					Root-weight ratio				
Contrast	Estimate	SE	df	t-ratio	p	Estimate	SE	df	t-ratio	p
CTRL - HTG	-0.08	0.024	610	-3.351	**0.0025**	-0.0578	0.019	610	-2.908	**0.0105**
CTRL - HMG	-0.0613	0.028	610	-2.166	*0.0779*	-0.0337	0.022	610	-1.53	0.2776
HTG - HMG	0.0186	0.009	610	2.063	*0.0985*	0.0242	0.006	610	3.798	**0.0005**

Supplementary Table 5 Pairwise post-hoc comparisons of the different microplastic distributions regarding the root-morphology traits specific root length (SRL), average root diameter and average link length, obtained using the "emmeans" package (Lenth, 2021). HTG refers to the heterogeneous plastic treatment, HMG to the homogeneous plastic treatment and CTRL to the control treatment.

	SRL					Root diameter					Link length				
Contrast	Estimate	SE	df	t-ratio	p	Estimate	SE	df	t-ratio	p	Estimate	SE	df	t-ratio	p
CTRL - HTG	0.2138	0.069	605	3.1	**0.0057**	-0.0715	0.0098	605	-7.285	**< 0.001**	0.1582	0.0523	605	3.026	**0.007**
CTRL - HMG	0.1796	0.073	605	2.466	**0.037**	-0.0463	0.0112	605	-4.147	**< 0.001**	0.1318	0.0459	605	2.868	**0.0119**
HTG - HMG	-0.0342	0.044	605	-0.773	0.7195	0.0252	0.0097	605	2.604	**0.0255**	-0.0264	0.0157	605	-1.687	0.211

Supplementary Table 6 Pairwise post-hoc comparisons of different microplastic distributions regarding root-foraging responses, obtained using the "emmeans" package (Lenth, 2021). HTG refers to the heterogeneous plastic treatment, HMG to the homogeneous plastic treatment and CTRL to the control treatment.

	CTRL - HTG					CTRL - HMG					HTG - HMG				
	Estimate	SE	df	t-ratio	p	Estimate	SE	df	t-ratio	p	Estimate	SE	df	t-ratio	p
FI root biomass	-0.345	0.039	626	-8.852	**< 0.001**	-0.007	0.019	626	-0.935	0.935	0.338	0.039	626	8.63	**< 0.001**
FI SRL	-0.063	0.017	605	-3.719	**< 0.001**	-0.017	0.019	605	-0.851	0.672	0.047	0.017	605	2.732	**0.018**
FI root length	-0.213	0.032	605	-6.702	**< 0.001**	0.007	0.021	605	0.322	0.944	0.219	0.037	605	5.999	**< 0.001**
FI root diameter	-0.045	0.011	621	-4.198	**< 0.001**	-0.002	0.006	621	-0.369	0.928	0.043	0.009	621	4.659	**< 0.001**
FI link length	-0.144	0.024	605	-5.965	**< 0.001**	0.005	0.008	605	0.67	0.781	0.149	0.028	605	5.268	**< 0.001**

Supplementary Table 7 Results of linear mixed-effects models testing patch-level effects of microplastic (MP) distribution treatments (control, heterogeneous, homogeneous) on plant-root morphology. Fixed-factor effects were assessed using log-likelihood ratio tests (Zuur et al., 2009). Log-likelihood ratios (LLR) are approximately χ^2-distributed. P values < 0.05 are indicated in bold.

		Root biomass		SRL		Root length		Root diameter		Link length	
Fixed effects	df	LLR	p	LLR	p	LLR	p	LLR	p	LLR	p
MP treatment	2	53.965	**< 0.001**	16.853	**< 0.001**	34.179	**< 0.001**	12.352	**0.002**	6.119	**0.047**
Patch	1	153.934	**< 0.001**	9.578	**0.002**	102.893	**< 0.001**	38.137	**< 0.001**	15.164	**< 0.001**
MP:Patch	2	250.92	**< 0.001**	11.027	**0.004**	131.95	**< 0.001**	57.527	**< 0.001**	35.217	**< 0.001**
Random effects		SD		SD		SD		SD		SD	
Family		0.03488		0.46119		4.79596		0.13387		0.33835	
Species		0.06286		0.31857		10.96738		0.13793		0.27339	
Sample		0.06202		0.39752		12.31821		0.11962		0.11367	
Residual		0.01539		0.37408		14.69482		0.08124		0.46957	

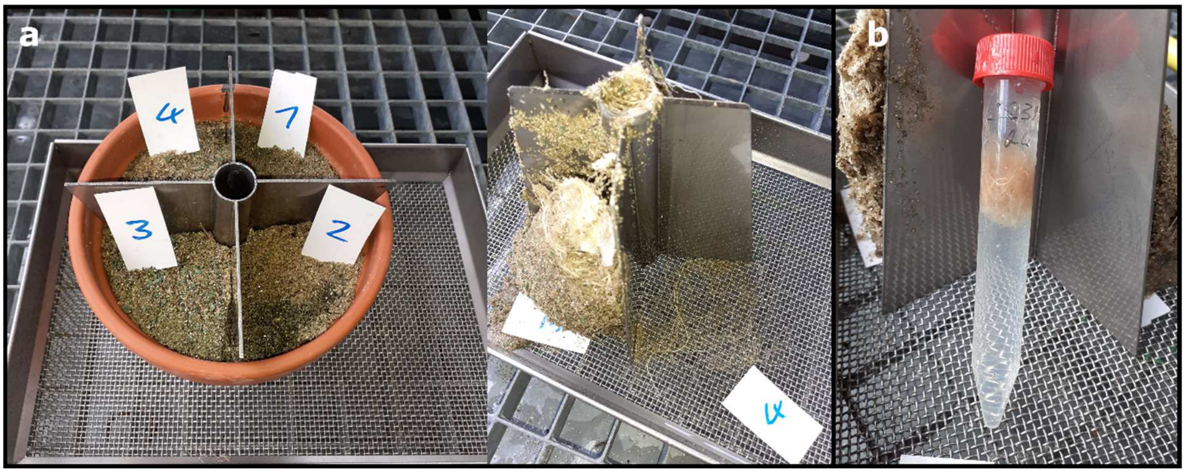

Supplementary Figure 1 Overview of the different steps of root separation and washing. a: Substrate and roots were cut according to previously assigned quarters (plastic-, or control-patches) and the central core, using a soil corer with four knife blades on its outside at 90° angles (left). The roots included in each quarter (and the central part) were cleaned from substrate and plastic residues (right). b: Roots for the root-morphology analysis were stored in water-filled plastic tubes and were kept at approx. 10°C until they were scanned. Afterwards, they were also dried and weighed.

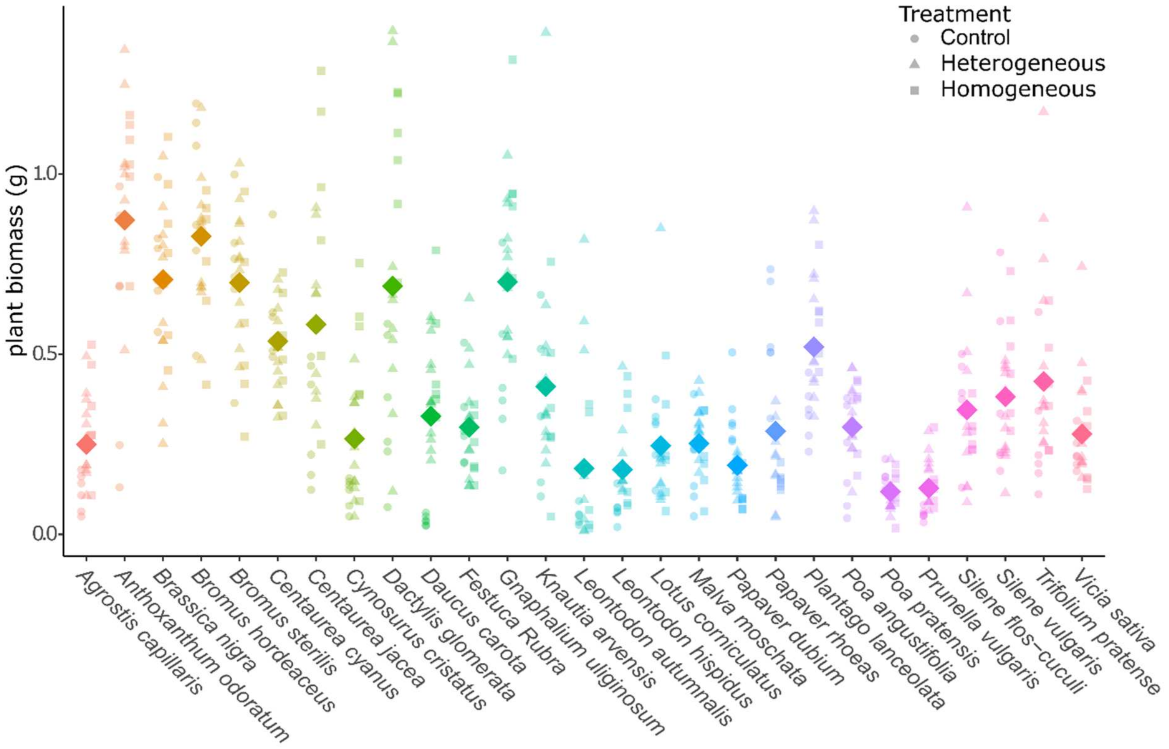

Supplementary Figure 2 Biomass variation among and within species. Different shapes indicate treatments, large diamonds represent average species biomass.

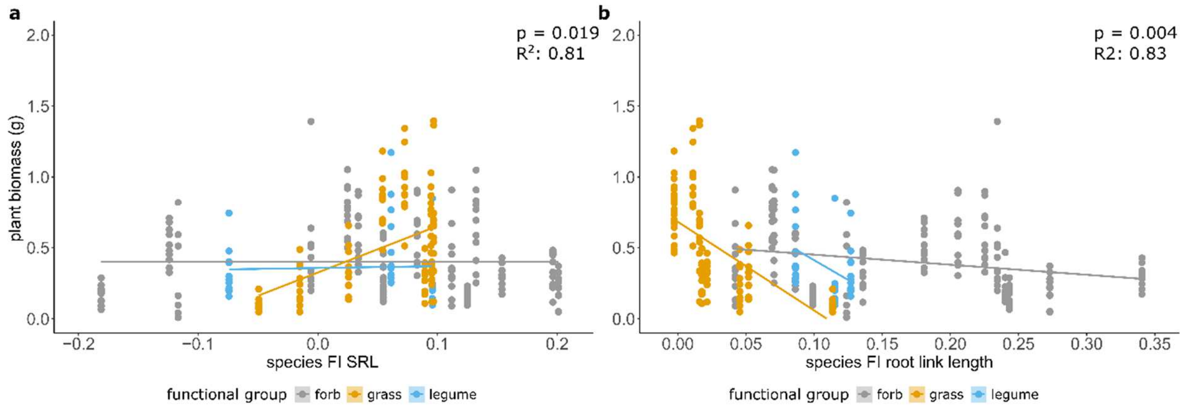

Supplementary Figure 3 Interactive effects of foraging response (species FI) and functional group affiliation on plant performance. Colors represent forbs (grey), grasses (gold) and legumes (blue). Species foraging index (FI) values refer to the average FI of each species. P values refer to the interaction between functional group and average species FI and were obtained using likelihood-ratio tests. R^2 values represent the conditional R^2 of the respective model.

References

Lenth, R. V. (2021). emmeans: Estimated Marginal Means, aka Least-Squares Means. R package version 1.7.1-1. https://CRAN.R-project.org/package=emmeans

Zuur, A. F., Ieno, E. N., Walker, N. J., Saveliev, A. A., & Smith, G. M. (2009). *Mixed effects models and extensions in ecology with R*. Springer. https://doi.org/10.1007/978-0-387-87458-6
